# Rack1‐mediated ferroptosis affects hindgut development in rats with anorectal malformations: Spatial transcriptome insights

**DOI:** 10.1111/cpr.13618

**Published:** 2024-03-25

**Authors:** Chen‐Yi Wang, Mu‐Yu Li, Si‐Ying Li, Xiao‐Gao Wei, Nai‐Xuan Dong, Shu‐Ting Liu, Zheng‐Wei Yuan, Bo Li, Agostino Pierro, Xiao‐Bing Tang, Yu‐Zuo Bai

**Affiliations:** ^1^ Department of Pediatric Surgery Shengjing Hospital of China Medical University Shenyang China; ^2^ Key Laboratory of Health Ministry for Congenital Malformation Shengjing Hospital of China Medical University Shenyang China; ^3^ Division of General and Thoracic Surgery The Hospital for Sick Children Toronto Canada

## Abstract

Anorectal malformation (ARM), a common congenital anomaly of the digestive tract, is a result of insufficient elongation of the urorectal septum. The cytoplasmic protein Receptor of Activated C‐Kinase 1 (Rack1) is involved in embryonic neural development; however, its role in embryonic digestive tract development and ARM formation is unexplored. Our study explored the hindgut development and cell death mechanisms in ARM‐affected rats using spatial transcriptome analysis. We induced ARM in rats by administering ethylenethiourea via gavage on gestational day (GD) 10. On GDs 14–16, embryos from both normal and ARM groups underwent spatial transcriptome sequencing, which identified key genes and signalling pathways. *Rack1* exhibited significant interactions among differentially expressed genes on GDs 15 and 16. Reduced Rack1 expression in the ARM‐affected hindgut, verified by Rack1 silencing in intestinal epithelial cells, led to increased P38 phosphorylation and activation of the MAPK signalling pathway. The suppression of this pathway downregulated Nqo1 and Gpx4 expression, resulting in elevated intracellular levels of ferrous ions, reactive oxygen species (ROS) and lipid peroxides. Downregulation of Gpx4 expression in the ARM hindgut, coupled with Rack1 co‐localisation and consistent mitochondrial morphology, indicated ferroptosis. In summary, *Rack1*, acting as a hub gene, modulates ferrous ions, lipid peroxides, and ROS via the P38‐MAPK/Nqo1/Gpx4 axis. This modulation induces ferroptosis in intestinal epithelial cells, potentially influencing hindgut development during ARM onset.

## Graphical abstract

Spatial transcriptome sequencing of normal and anorectal malformation (ARM) rat embryos revealed that *Rack1*, as a hub gene, was decreased in the ARM hindgut on GD15 and GD16, and increased intracellular ferrous ions, reactive oxygen species, and lipid peroxides through the P38/Nqo1/Gpx4 axis, inducing ferroptosis in intestinal epithelial cells, which may affect the hindgut development in rat with ARM.

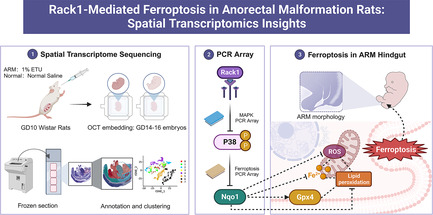

## 1 INTRODUCTION

Anorectal malformation (ARM) is a common congenital anomaly of the digestive tract occurring in children, with an incidence rate of 1 in 5000.^1^ The etiology of ARM remains elusive, with both genetic and environmental factors implicated in its pathogenesis. Recent studies have highlighted the pivotal role of genetic regulation in this process.^2^, ^3^ Typically occurring during weeks 4–8 of gestation, ARM is predominantly diagnosed postnatally and usually requires surgical intervention as treatment.^4^ However, the long‐term post‐surgery outcomes in patients with ARM are suboptimal, often leading to complications, such as faecal incontinence and chronic constipation. These complications have a substantial impact on the quality of life and psychosocial development of affected children.^5^, ^6^

Owing to the challenges in obtaining early‐pregnancy clinical samples, ethylenethiourea (ETU)‐induced rat models have become a widely used method for studying ARM, as they closely resemble the human ARM phenotype.^7^, ^8^ During rat embryonic development, gestation days (GDs) 14–16 are pivotal for the maturation of the cloaca, with a focus on the hindgut, urorectal septum (URS) and cloacal membrane (CM). A significant developmental milestone occurs on GD 15, characterised by the successful separation of the hindgut from the urethra. By GD 14, the tail groove forms in normal rat embryos, reducing the size of the cloacal duct that connects the hindgut to the urogenital sinus, with the distal end of the hindgut nearing the tail groove. On GD 15, the epithelial cells at the tip of the URS converge with those near the dorsal CM, effectively separating the hindgut from the urethra and connecting the tail groove to the hindgut. By GD 16, the anal membrane (AM) ruptures, forming the anus and establishing complete hindgut communication with the external environment, thereby completing normal anorectal development. Conversely, embryos with ARM exhibit an underdeveloped dorsal CM and URS, with failed URS–CM fusion, leading to the hindgut remaining connected to the urethra, often resulting in a rectourethral fistula. Furthermore, many ARM embryos exhibit short or absent tails.^9^, ^10^, ^11^

ARM encompasses a range of congenital anomalies regulated by complex signalling pathways, highlighting the need for further research into the key genes and pathways involved. The intricate anatomy of the cloaca presents challenges for conventional sequencing methods during library preparation, which can hinder the identification of differentially expressed genes (DEGs) in specific anatomical locations.^12^ Spatial transcriptomics technology facilitates accurate gene screening, enabling the detection of spatiotemporal expression patterns and interactions, and ultimately avoiding interference between distinct cloacal structures. This approach sheds light on the molecular functions within specific structures and may facilitate dynamic investigations into ARM development.

Abnormal cell death in the URS and hindgut regions is a key factor contributing to the failure of URS and CM fusion in rat embryos.^13^, ^14^ This finding has spurred investigations into cell death mechanisms within the cloacal region. Our spatial transcriptomic analysis identified the occurrence of ferroptosis, an iron‐dependent programmed cell death, characterised by the accumulation of reactive oxygen species (ROS) and lipid peroxidation products, within the ARM hindgut.^15^ We hypothesise that this process is regulated by Receptor of Activated C‐Kinase 1 (Rack1), a cytoplasmic protein shares similarities with guanine nucleotide‐binding proteins.^16^ It plays diverse roles in cell growth, differentiation, signalling and immune responses and is potentially significant in embryonic neural development.^17^ However, previous studies have not reported the role of *Rack1* in the regulation of ferroptosis. Therefore, its role in embryonic digestive tract development and ARM formation requires further exploration.

In this study, we conducted spatial transcriptomic sequencing analysis on both normal and ARM rat embryos during GDs 14–16, obtaining gene transcription information with specific anatomical relevance. We used bioinformatics methods to identify key genes in the hindgut region and assessed the activity of relevant signalling pathways within the cloacal area. Furthermore, we identified ferroptosis in the hindgut of ARM embryos and investigated the potential regulatory mechanisms via the Rack1 and P38/Nqo1 pathways. These investigations provide deeper insights into the molecular mechanisms involved in ARM and hindgut development, thereby contributing to the enhancement of prenatal diagnosis and therapeutic approaches.

## 2 METHODS

### 2.1 ARM rat model preparation

Healthy female Wistar rats (10–12 weeks old; 200–230 g; HFK Bioscience Co., Ltd., Beijing, China) were selected and maintained under specific pathogen‐free conditions. Sexually mature and healthy male Wistar rats were also selected, and females and males were co‐housed overnight at a 3:1 ratio. The following morning, vaginal smears were collected from female rats to ascertain the presence of sperm. Females with sperm present were marked as GD 0. Pregnant females were then housed individually and randomly assigned to either the ARM or normal groups. In the ARM group, pregnant rats were orally administered 1% ETU (Sigma‐Aldrich; Merck Millipore, Darmstadt, Germany) at a dose of 125 mL/kg on GD 10. In contrast, pregnant rats in the control group received equivalent doses of normal saline. From GDs 14–16, the pregnant rats were anaesthetised using isoflurane inhalation combined with an intraperitoneal injection of lidocaine, followed by a caesarean section to retrieve the embryos. All animal experiments conducted in this study were approved by the Ethics Committee of Shengjing Hospital, China Medical University (Approval No. 2020PS357K).

### 2.2 Embedding of frozen tissue

Following caesarean section, the retrieved embryos were immediately placed on cold normal saline to remove surface blood. Thereafter, embryos were positioned in a horizontal, sagittal orientation within an embedding box containing a pre‐chilled optimal cutting temperature (OCT) compound (Sakura, Nagano, Japan). The embedding box was quickly immersed in pre‐chilled isopentane (Sigma‐Aldrich) for 1 min. Upon complete solidification of the OCT, the specimens were carefully transferred and stored in a freezer maintained at a temperature of −80°C.

### 2.3 Pathway RespOnsive GENes (PROGENy) and non‐negative matrix factorisation (NNMF) algorithms

Pathway activity analysis was conducted using the ‘PROGENy’ package (version 1.12.0) in R software (version 4.0.3; R Foundation for Statistical Computing, Vienna, Austria). To address spatial transcriptomic standardisation, the PROGENy standardisation parameter was set to FALSE. The *ScaleData* function from the ‘Seurat’ R package was used for normalisation. Spatial transcriptome data were enriched using PROGENy activity scores, which were then scaled to obtain spot‐specific score data. These scores were integrated with clustering results, and pathway‐specific mean activity scores were calculated for each cluster. The resulting dataset was used to generate a heatmap using the R package ‘heatmap’. For dimensionality reduction, the NNMF approach facilitated by the ‘STutility’ R package was used, achieving results comparable to those obtained via principal component analysis. The correlation between the PROGENy activity score values and dimensionality reduction outcomes was determined using Pearson's method. A correlation heatmap was subsequently created using the R package ‘heatmap’.

### 2.4 Protein–protein interaction network

The designated gene set was entered into the ‘multiple proteins’ search field of the STRING database (https://string-db.org) for comprehensive analysis. The network's individual nodes were concealed, and the acquired data were extracted in tab‐separated values (TSV) format. This TSV file was then imported into the Cytoscape software (version 3.9.1), where the integrated CytoNCA tool was used to rank the dataset based on betweenness centrality (BC) values and to construct a circular Protein–protein interaction (PPI) network diagram.

### 2.5 Paraffin embedding and sectioning

The embryos were thoroughly rinsed with phosphate‐buffered saline (PBS) to remove surface blood and then immersed in a 4% paraformaldehyde solution at 25°C for 16–24 h. After rinsing with running water for 8 h, the samples were dehydrated using a series of graded ethanol solutions. Thereafter, the embryos were immersed in xylene for 1 h and embedded in liquid paraffin following a 3‐h soaking period. The wax blocks were then carefully sectioned into 3.5 μm‐thick slices along the mid‐sagittal plane using a precision section cutter, after which they were kept in an incubator at 65°C for 2–3 h.

### 2.6 Immunofluorescence staining

Paraffin sections that clearly and comprehensively displayed the urethra, hindgut, URS and rectourethral fistulas were selected for staining. The sections first underwent deparaffinisation and antigen retrieval in water. Blocking was conducted using serum (ZSGB‐BIO, Beijing, China) at 25°C for 1 h, followed by incubation with primary antibodies for 16 h at 4°C. The following primary antibodies were used: Anti‐Rack1 (1:100) and anti‐Uba52 (1:100) purchased from Abcam (Cambridge, UK); Anti‐Tpt1 (1:100), anti‐Npm1 (1:100), anti‐Eef1b2 (1:100), anti‐Erk1/2 (1:100), anti‐Mapk14 (1:100) and anti‐Gpx4 (1:100) purchased from Proteintech (Wuhan, China); Anti‐Erk3 (1:100) purchased from Abmart (Shanghai, China). Following a series of PBS washes, the sections were incubated for 1 h at 25°C in the absence of light with the following fluorescent secondary antibodies: Goat Anti‐Rabbit 488 (1:100), Goat Anti‐Rabbit 647 (1:100) and Goat Anti‐Mouse 488 (1:100), all purchased from Proteintech. Following additional PBS washes, DAPI staining solution (Servicebio Technology, Wuhan, China) was applied, and the sections were incubated for 10 min. Finally, the sections were mounted using an anti‐fluorescence attenuation medium (Solarbio, Beijing, China) and images were captured using a laser‐scanning confocal microscope (LSM 880, Zeiss, Oberkochen, Germany).

### 2.7 Polymerase chain reaction array assay

RNA was extracted from cell samples and reverse transcribed into cDNA, which was then diluted and mixed to a final volume of 100 μL. The cycle threshold (Ct) value of the reference gene in the original cDNA solution of the cell samples was maintained at 17. Prior to use, a polymerase chain reaction (PCR) array plate (WcGene Biotech, Shanghai, China) was centrifuged at 2000 rpm for 20 s. In accordance with the manufacturer's instructions, cDNA and SYBR mix (Takara Bio, Shiga, Japan) suspension was prepared, thoroughly mixed, and 9 μL of the mixture was added to each well. The plate was centrifuged at 2000 rpm for 20 s before being processed through PCR. The PCR setup consisted of a 10 μL reaction system, and the reaction conditions were set according to the manufacturer's instructions.

### 2.8 Utilisation of inhibitors and inducers

To induce ferroptosis, cells were treated with 10 μM Erastin (APExBIO Technology, Houston, TX, USA) for 24 h. Ferrostatin‐1 (Fer‐1; APExBIO Technology) was used at a concentration of 100 nM to inhibit ferroptosis for 24 h. Suppression of P38‐MAPK activity was achieved by treating cells with 520 nM doramapimod (MedChemExpress, MCE, Rahway, NJ, USA) for 24 h. To induce Nqo1 activity, cells were treated with 2‐HBA (MCE) at a concentration of 0.6 μM for 24 h. The siRNA of Rack1 (RiboBio, Guangzhou, China) was used at a concentration of 80 nM, with a treatment time of 24–36 h.

### 2.9 Intracellular ferrous ion detection

Intracellular ferrous iron was detected using the FerroOrange fluorescent probe (Dojindo, Beijing, China). To prepare a 1 mmol/L FerroOrange solution, 35 μL of dimethyl sulfoxide (DMSO; Beyotime Biotechnology, Shanghai, China) was added to a tube containing 24 μg FerroOrange. This was then diluted with Hank's Balanced Salt Solution (HBSS; Servicebio Technology) to generate a 1 μmol/L working solution. Cells were cultured in confocal culture dishes (Biosharp, Hefei, China), treated with a working solution of FerroOrange, and incubated at 37°C in a 5% CO_2_ incubator for 30 min. Finally, observations were made using a laser‐scanning confocal microscope at an excitation wavelength of 561 nm.

### 2.10 Intracellular lipid peroxide detection

A Liperfluo fluorescence probe (Dojindo) was used to assess the intracellular lipid peroxide levels. A 1 mmol/L Liperfluo solution was prepared by combining 60 μL of DMSO with 50 μg Liperfluo. Following treatment, cells were removed from the culture medium and washed with HBSS. A working solution of Liperfluo was prepared with a final concentration of 10 μmol/L by diluting in serum‐free culture medium. This solution was added to the cells, which were then incubated at 37°C for 30 min. The cells were observed using a laser‐scanning confocal microscope at an excitation wavelength of 488 nm.

### 2.11 ROS detection

To assess intracellular ROS levels, a 2′,7′‐Dichlorodihydrofluorescein diacetate probe (DCFH‐DA; Servicebio Technology) was used. The DCFH‐DA probe was diluted in a serum‐free cell culture medium at a ratio of 1:3000 to prepare the working solution. Before measurement, the cell culture medium was aspirated, and the cells were treated with the DCFH‐DA working solution, and incubated at 37°C for 30 min. After incubation, the cells were washed twice with PBS and observed under a laser‐scanning confocal microscope at an excitation wavelength of 488 nm.

### 2.12 Protein preparation and western blot

The total protein lysis buffer consisted of RIPA buffer (Beyotime Biotechnology), 1% phenylmethanesulfonyl fluoride (PMSF; Beyotime Biotechnology) and a phosphatase inhibitor (APExBIO Technology). The lysate was boiled for 5 min in loading buffer (Epizyme Biomedical Technology, Shanghai, China) to denature the proteins. Concentrated sodium dodecyl sulphate‐polyacrylamide gel electrophoresis (SDS‐PAGE) gels (10%; Epizyme Biomedical Technology) were prepared according to the manufacturer's instructions. Electrophoresis was conducted at 100 V for 100 min, followed by protein transfer to a polyvinylidene difluoride (PVDF) membrane (Millipore, Burlington, MA, USA). The PVDF membrane was blocked for 15 min using a fast‐blocking solution (Epizyme Biomedical Technology), and subsequently incubated with primary antibodies overnight at 4°C. After washing the membrane with TBST solution, it was incubated with secondary antibodies at room temperature for 2 h. Chemiluminescent detection was performed using Super Signal Chemiluminescent Substrate (Thermo Fisher Scientific, Waltham, MA, USA) and visualised using an Amersham Imager 680 (AI 680; General Electric, Boston, MA, USA). Band density was quantified using ImageJ software (http://fiji.sc/). The antibodies used were as follows: Anti‐Rack1 (1:500), Anti‐Nqo1 (1:10000), and Anti‐Gpx4 (1:3000) purchased from Abcam; Anti‐β‐actin (1:2000), Anti‐Phospho‐P38 (1:1000), Anti‐P38 (1:1000), Goat Anti‐Rabbit secondary antibody (1:5000) and Goat Anti‐Mouse secondary antibody (1:5000) purchased from Proteintech.

### 2.13 Cell viability assay

Cells were plated in 96‐well plates for treatment. After aspirating the culture medium and washing with PBS, the wells were replenished with 10% Cell Counting Kit‐8 (CCK‐8; APExBIO Technology) dissolved in a complete medium. Following 2 h of incubation at 37°C in a CO_2_ incubator, the absorbance at 450 nm was measured using a Tecan Infinite 200 microplate reader (Tecan Group, Männedorf, Switzerland).

### 2.14 Cell toxicity assay

A lactate dehydrogenase (LDH) Cytotoxicity Assay Kit (Servicebio Technology) was used for quantitative cell toxicity analysis. Prior to analysis, the culture medium was removed, and the cells were washed with PBS. Thereafter, 120 μL of cell lysis working solution was added, and cells were incubated in a CO_2_ incubator for 30 min. Next, 80 μL of lysate supernatant was transferred to a new 96‐well plate. Following the kit instructions, 80 μL of LDH detection working solution was added per well. After 30 min of light‐protected incubation, the absorbance was measured at 490 nm using a microplate reader (Tecan Group).

### 2.15 Application of online plotting software

The diagrams in Figures 4A,B and 6A,B were generated using an online microbioinformatics tool at http://www.bioinformatics.com.cn/. Figure 7 was created using Biorender software (https://www.biorender.com/).

### 2.16 Statistical analysis

Statistical analyses were performed using GraphPad Prism software (version 9.4.1; La Jolla, CA, USA). Student's *t*‐tests were used to determine the statistical differences between two groups. All values are expressed as mean ± standard deviation. Experiments were performed in triplicate, and significance was set at *p* < 0.05.

## 3 RESULTS

### 3.1 Spatial transcriptome sequencing sample setup and anatomical annotation clustering

**FIGURE 1:**
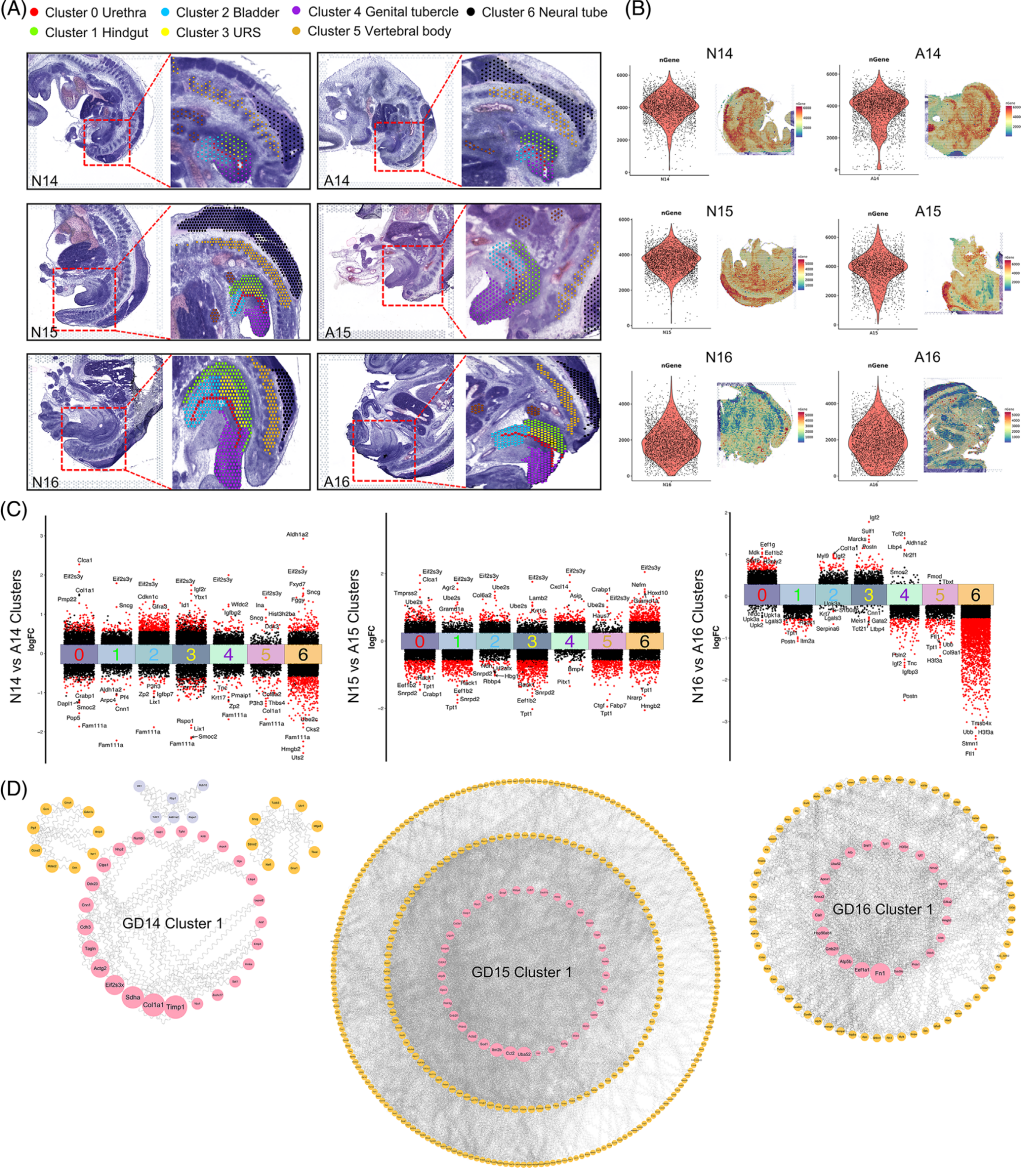
Morphology of sample sections and hub gene analysis. (A) Histological images of rat embryo mid‐sagittal sections from GDs 14–16 stained with haematoxylin & eosin (HE), used for spatial transcriptome sequencing. The red box indicates the region selected for anatomical annotation and dimensionality reduction clustering, and is divided into seven clusters based on different colours. (B) The average number of genes captured per patch. (C) Volcano plot of differentially expressed genes (DEGs) across seven clusters in embryos from GDs 14–16. Genes upregulated and downregulated in the ARM group are shown above and below the plot, respectively. (D) PPI analysis results for DEGs in the hindgut region of embryos from GDs 14–16. Hub genes with higher connectivity are depicted within the pink circle. A, anorectal malformations group; ARM, anorectal malformations; GD, gestational day; N, normal group; PPI, protein–protein interaction; URS, urorectal septum.

In this study, we used normal Wistar rat embryos on GDs 14–16 (referred to as N14–16) and ETU‐induced ARM embryos on GDs 14–16 (referred to as A14–16) to conduct spatial transcriptome sequencing. Six frozen mid‐sagittal sections of rat embryos, each 10‐μm thick, were prepared. These sections included both the urethral and hindgut layers, providing a comprehensive view of cloacal development at specific time points (Figure 1A). Using the 10× Genomics Visium spatial transcriptome technology, followed by tissue permeabilisation, cDNA synthesis and library construction, we successfully captured visual gene transcription data, predominantly from the cloacal region, in both normal and ARM embryos. To ensure comparability and consistency across various section samples from the same region, we performed anatomical clustering annotation, yielding seven distinct clusters. These clusters encompassed five specific zones (clusters 0–4) within the cloacal region, namely the urethra, hindgut, bladder, URS and genital tubercle. Additionally, two clusters (clusters 5 and 6) corresponded to the vertebral body and neural tube region (Figure 1A). On average, each anatomical area covered 3005 spots and each spot captured 16,905 genes (Figure 1B).

### 3.2 Screening of DEGs between the normal and ARM groups

The criteria for identifying DEGs were established as an absolute log_2_ fold change (|log_2_FC|) ≥ 0.58 and a significance level of *p* < 0.05. This screening led to the creation of differential volcano plots to illustrate alterations in gene expression. Within the context of hindgut development on GDs 14, 15 and 16, we respectively identified 91, 439 and 119 genes that exhibited distinct expression profiles at these time points (Figure 1C). Notably, *Rack1* (*Gnb2l1*) exhibited reduced expression within the hindgut of the ARM group on GDs 15 and 16. To further explore the potential molecular interactions, we performed a comprehensive PPI analysis. By leveraging BC values as a ranking criterion, we highlighted the most significant differentially expressed hindgut genes (Figure 1D). Hub genes, denoted by pink nodes within the circular arrangement, included *Uba52*, *Rack1* and *Tpt1*, each with a notable centrality within the gene network during GDs 15 and 16 of hindgut development. Notably, *Rack1* demonstrated exceptional connectivity, securing its position among the top five genes in the PPI networks on both GDs 15 and 16.

### 3.3 Validation of partial DEGs in the hindgut on GD 15

**FIGURE 2:**
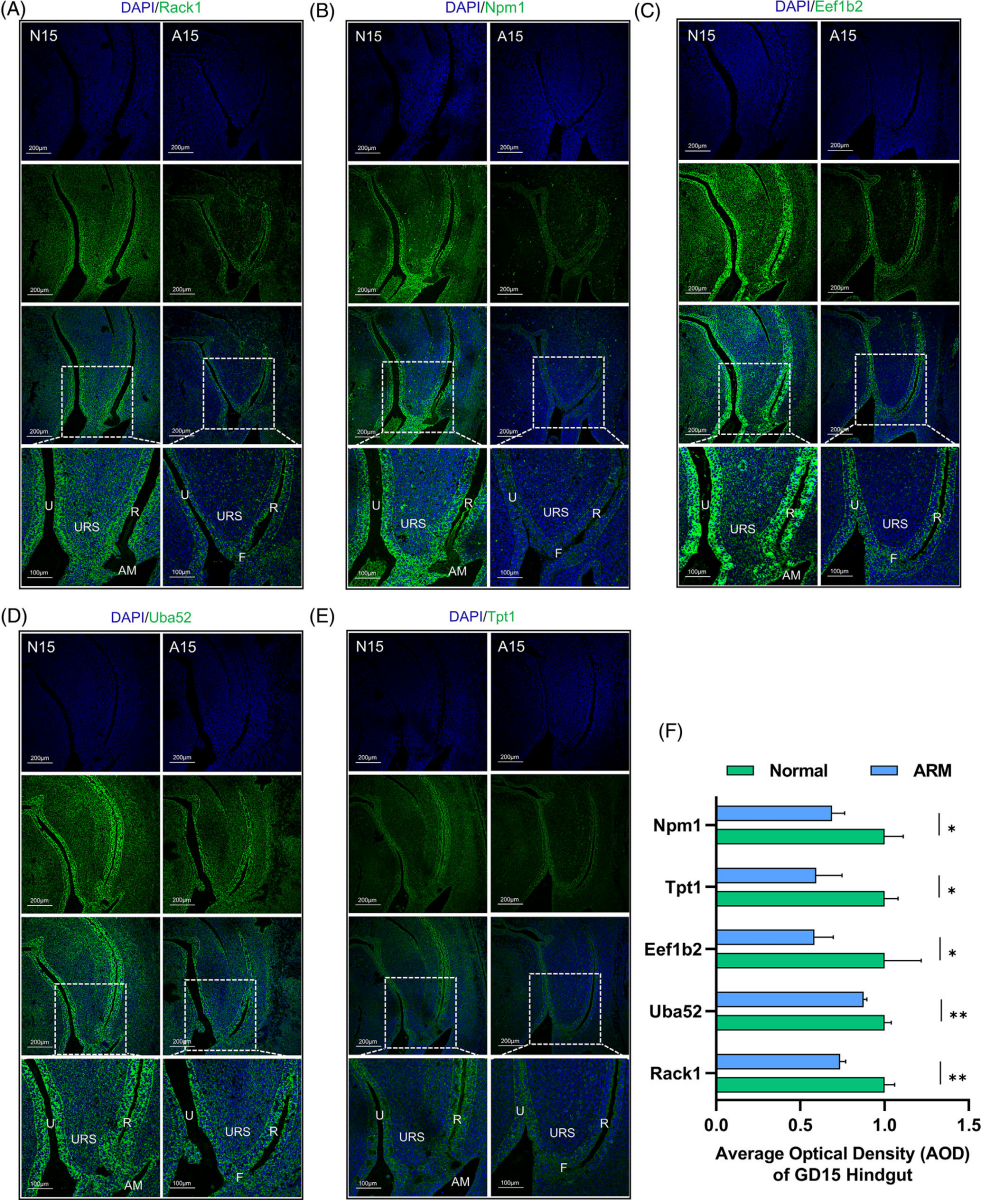
Spatial localisation and semi‐quantitative analysis of hub genes. Immunofluorescence staining of (A) Rack1 protein, (B) Npm1 protein, (C) Eef1b2 protein, (D) Uba52 protein and (E) Tpt1 protein in the GD 15 hindgut region. Green fluorescence indicates positive expression, and the white boxes represent the selected magnified areas. (F) Quantification and statistical analysis of the AOD in the GD15 hindgut region. A, anorectal malformations group; AM, anal membrane; AOD, average optical density; F, rectourethral fistula; N, normal group; GD, gestational day; R, rectum; U, urethra; URS, urorectal septum. Scale bar, 200 and 100 μm; *p < 0.05; **p < 0.01.

Figure 2A–E provides a comprehensive illustration of the protein expression patterns and localisations of the top five genes: *Rack1*, *Npm1*, *Eef1b2*, *Uba52* and *Tpt1*, in the cloacal region of GD15 sections. These figures were obtained using immunofluorescence staining. Notably, these five proteins exhibited site‐specific expression within both the urethral and hindgut epithelium of the N15 cloacal region. Apart from Tpt1, the other four proteins exhibited elevated expression levels within the AM region. Furthermore, these proteins were observed to be discretely distributed within the lower extremity of the URS and the interstitial vicinity of the hindgut. In comparison, a decline in the expression of these five proteins was evident within the urethral and hindgut epithelium in the cloacal region of A15, accompanied by reduced expression levels at the site of the rectourethral fistula (*p* < 0.05). Figure 2F presents a semi‐quantitative evaluation of the average optical density (AOD), specifically within the hindgut domain.

### 3.4 PROGENy algorithms predict concentrated enrichment of MAPK signalling pathway in the hindgut

**FIGURE 3:**
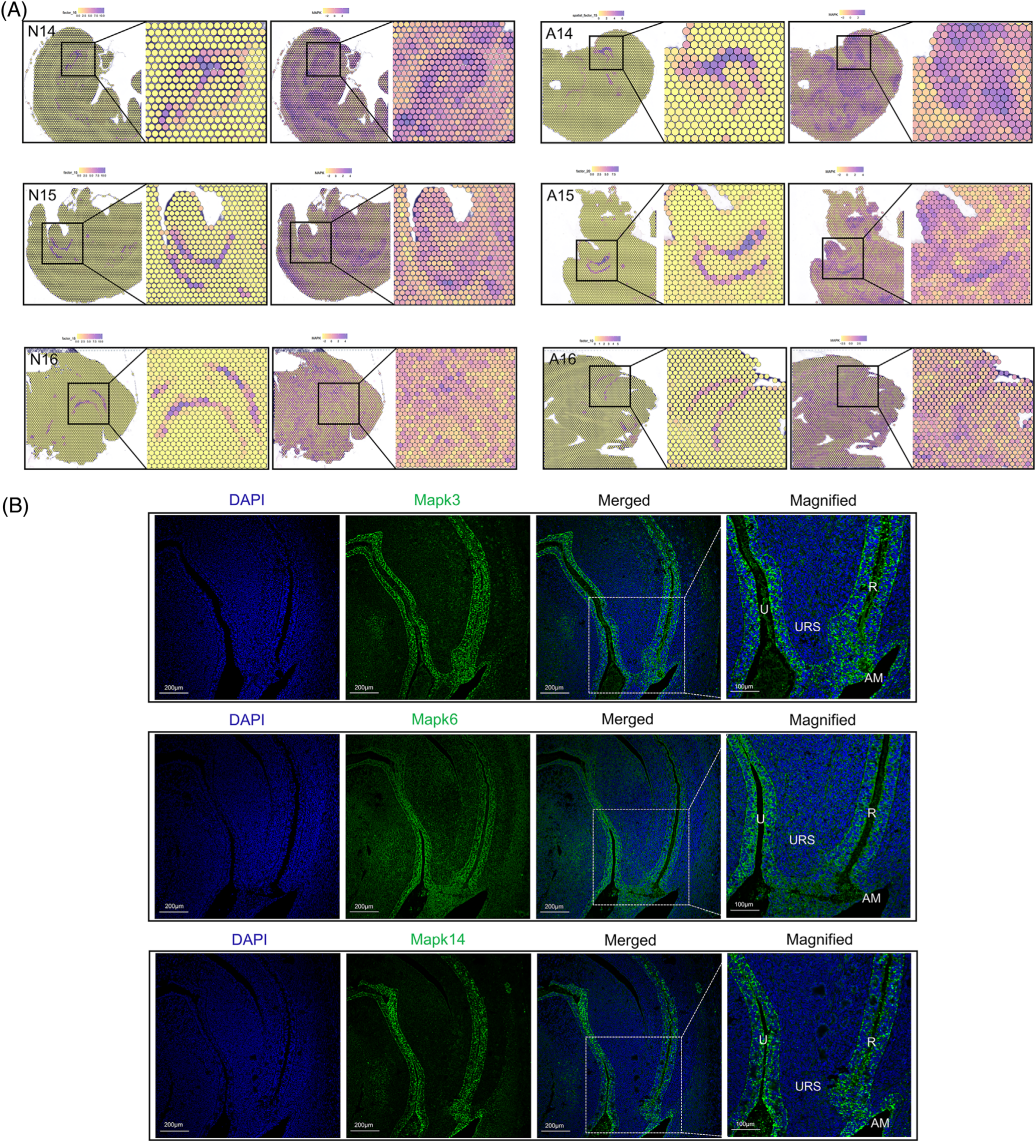
Prediction of signalling pathway activity based on cloacal spatial location. (A) NNMF clustering in the urethra/hindgut regions and the enrichment localisation of MAPK signalling pathway in six samples. (B) Spatial expression and localisation and immunofluorescence staining of Mapk3, Mapk6, and Mapk14. A, anorectal malformations group; AM, anal membrane; GD, gestational day; N, normal group; NNMF, Non‐negative matrix factorisation; R, rectum; U, urethra; URS, urorectal septum. Scale bar, 200 and 100 μm.

The PROGENy algorithm was used to evaluate the activity of signalling pathways through changes in downstream gene expression. We obtained scores for the activity levels of different pathways in the samples from GDs 14–16 in the normal and ARM groups. In the unsupervised clustering analysis of the hindgut region (N14‐factor 16, A14‐factor 15, N15‐factor 15, A15‐factor 20, N16‐factor 18 and A16‐factor 10) using NNMF, the PROGENy algorithm revealed a strong positive correlation with the MAPK signalling pathway, indicating a significant enrichment of MAPK signalling in the hindgut (Figure 3A). The localised expression patterns of 12 member molecules from the Erk, P38, JNK and Erk5 families were extracted from these regions. Specifically, Mapk3, Mapk6 and Mapk14 were predominantly enriched in the cloacal region. Immunofluorescent validation of these proteins confirmed their specific expression in the urethral and hindgut regions, with comparatively weaker expression in the URS and interstitial areas, which was consistent with the PROGENy predictions (Figure 3B).

### 3.5 Exploring the spatiotemporal expression patterns of Gpx4 through cell death gene mapping

**FIGURE 4:**
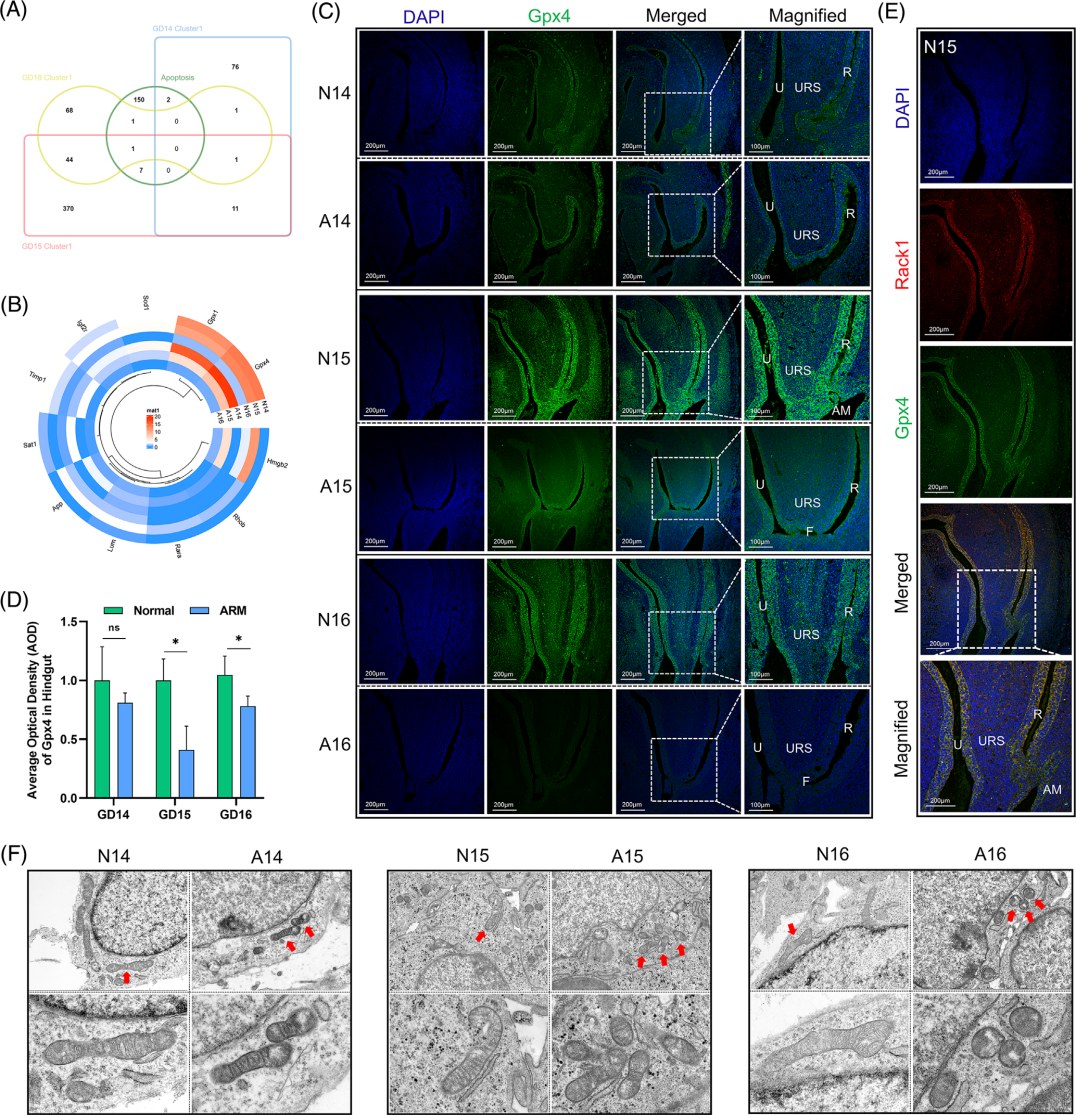
Spatiotemporal expression patterns of Gpx4 and mitochondrial morphology in the hindgut. (A) Venn diagram depicting the intersection of differentially expressed cell death genes in cluster 1 on GDs 14–16. (B) Heatmap displaying the expression of selected cell death genes in cluster 1. (C) Immunofluorescence staining for the localisation of Gpx4 in the hindgut on GDs 14–16. (D) Quantification of Gpx4 fluorescence intensity in the hindgut region (AOD analysis). (E) Immunofluorescence double staining showing co‐localisation of Rack1 and Gpx4. (F) TEM observations of mitochondrial morphological changes in the hindgut on GDs 14–16. A, anorectal malformations group; AM, anal membrane; AOD, average optical density; F, rectourethral fistula; GD, gestational day; N, normal group; R, rectum; U, urethra; TEM, Transmission electron microscopy; URS, urorectal septum. Scale bar, 200 μm and 100 μm; *p < 0.05.

In the gene set enrichment analysis (GSEA) database, the cell death gene set contained 161 genes, of which seven cell death‐related genes were obtained from the intersection of the gene set with the DEGs in cluster 1 on GD 15 (Figure 4A). The circular heat map illustrates the expression profiles of cell death genes within the same area as the six samples from cluster 1. Notably, *Gpx4*, a key member of the glutathione peroxidase family, was found to be downregulated in cluster 1 on GD 15 (*p* < 0.05; Figure 4B). Immunofluorescence staining revealed prominent localisation of the Gpx4 protein in the epithelium of the urethra and hindgut, as well as in the AM region, with a relatively lighter distribution observed in the URS. A reduction in Gpx4‐positive signalling was observed in the cloaca of the ARM group (Figure 4C). AOD analysis indicated a statistically significant reduction in Gpx4 expression in the hindgut on GDs 15 and 16 (*p* < 0.05; Figure 4D).

### 3.6 Co‐localisation of Rack1 and Gpx4, and observations of hindgut mitochondrial morphology

Double‐immunofluorescence staining of paraffin sections of N15 embryos revealed co‐localisation of the Rack1 and Gpx4 proteins within the urethra and hindgut epithelium. This result indicates the potential role of Rack1 in regulating ferroptosis in these tissues (Figure 4E). Subsequently, a microdissection was performed to obtain the hindgut tissues from both normal and ARM embryos. Transmission electron microscopy (TEM) revealed that cells in the ARM group exhibited reduced mitochondrial volume, increased mitochondrial density and decreased mitochondrial cristae. These observations are consistent with the mitochondrial morphological changes associated with the occurrence of ferroptosis (Figure 4F).

### 3.7 Spatiotemporal expression profile of Rack1 on GDs 14 and 16

**FIGURE 5:**
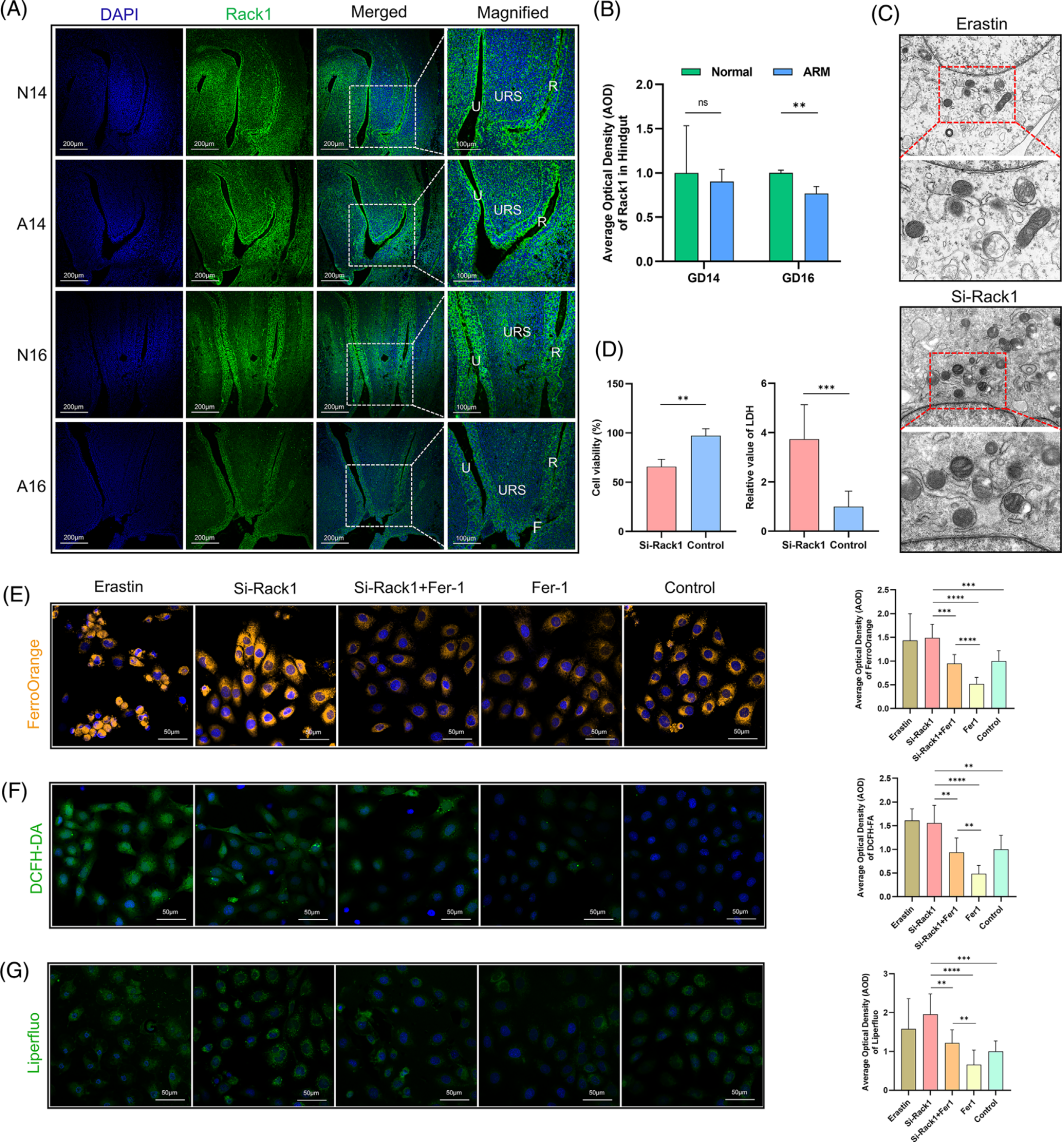
Rack1 mediates ferroptosis in intestinal epithelial cells. (A) Immunofluorescence staining for the localisation of Rack1 in the hindgut on GDs 14 and 16. (B) AOD analysis of Rack1 fluorescence intensity. (C) TEM observations of mitochondrial morphology in IEC‐6 cells after Rack1 and Erastin treatment. (D) CCK‐8 and lactate dehydrogenase (LDH) assays for measuring cellular viability and cytotoxicity levels, respectively. (E) FerroOrange probe detection of intracellular ferrous ion content. (F) DCFH‐DA probe detection of intracellular ROS levels. (G) Liperfluo probe detection of intracellular lipid peroxidation levels. Quantification and statistical analysis of cellular fluorescence intensity is shown on the right. A, anorectal malformations group; AOD, average optical density; F, rectourethral fistula; Fer‐1, Ferrostatin‐1; GD, gestational day; N, normal group; R, rectum; ROS, reactive oxygen species; U, urethra; TEM, Transmission electron microscopy; URS, urorectal septum; Scale bar, 200, 100 and 50 μm; **p < 0.01; ***p < 0.001; ****p < 0.0001.

Having previously described the localisation of the Rack1 protein in the cloacal region on GD 15 (Figure 2A), we further investigated its localisation in the cloacal region on GDs 14 and 16. In normal embryos (N14), Rack1‐positive signals were predominantly observed in the epithelial cells of the urethra and hindgut, with higher concentrations at the distal end of the hindgut. These signals were also scattered within the URS and mesenchymal cells. In ARM embryos (A14), Rack1‐positive cells were observed at the tip of the URS, hindgut, and urethral epithelium (Figure 5A). Comparative analysis revealed no significant difference in the AOD of Rack1‐positive signals between the N14 and A14 groups (Figure 5B). In normal rat embryos on GD 16 (N16), Rack1 expression was still prominent in the urethral and hindgut epithelia, but was limited in the URS and surrounding mesenchymal areas (Figure 5A). Notably, there was a decrease in fluorescence intensity in the hindgut region of A16 (*p* < 0.05), as depicted in Figure 5B, which illustrates the semi‐quantitative AOD results in the hindgut region.

### 3.8 Knockdown of Rack1 induces ferroptosis in intestinal epithelial cells

After si‐Rack1 transfection in IEC‐6 cells, the cellular ultrastructure was observed using TEM. The si‐Rack1 group exhibited intact cells and nuclear membranes, reduced mitochondrial size and increased mitochondrial density. Furthermore, mitochondrial cristae were either reduced or absent, resembling the features observed after erastin treatment (Figure 5C). Rack1 knockdown led to decreased cellular viability, as observed in the CCK‐8 assays, and increased cellular toxicity, as indicated by the LDH assays (*p* < 0.05; Figure 5D). Fluorescence analysis using the FerroOrange probe indicated elevated intracellular ferrous ion concentrations in the si‐Rack1 group, which decreased upon Fer‐1 treatment (*p* < 0.05; Figure 5E). Measurement of intracellular ROS levels using the DCFH‐DA probe revealed high ROS levels in the si‐Rack1 group, which decreased after Fer‐1 treatment (*p* < 0.05; Figure 5F). Liperfluo probe assessment revealed Fer‐1's ability to mitigate elevated lipid peroxide levels in the si‐Rack1 group (*p* < 0.05; Figure 5G). Fluorescence intensity quantification results are shown on the right.

### 3.9 Rack1 knockdown enhances P38 phosphorylation and modulates downstream Nqo1/Gpx4 expression

**FIGURE 6:**
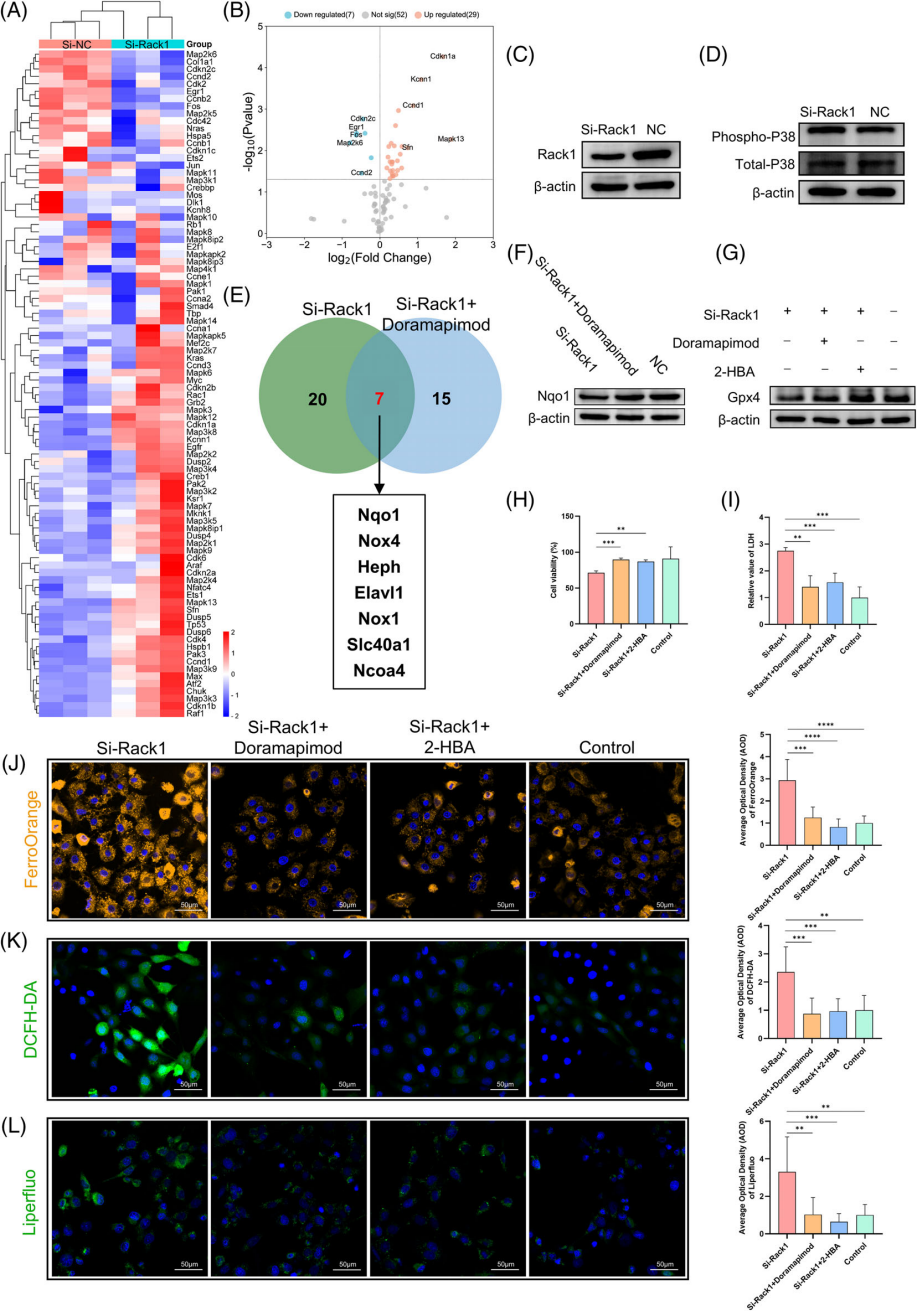
Screening of PCR arrays and Rack1/P38/Nqo1 axis in regulating ferroptosis. (A) Heatmap showing gene changes in the MAPK pathway PCR array after Rack1 knockdown. (B) Volcano plot displaying differentially upregulated and downregulated genes in the MAPK pathway PCR array after Rack1 knockdown. (C, D). Western blot indicating increased phosphorylation of P38 after Rack1 knockdown. (E) Downstream molecular targets screened by the ferroptosis gene PCR array in the si‐Rack1 and si‐Rack1 + Doramapimod groups. (F) Western blot showing Doramapimod rescue after the reduction of Nqo1 by Rack1 knockdown. (G) Western blot showing Doramapimod and 2‐HBA rescue after the reduction of Gpx4 by Rack1 knockdown. (H) CCK‐8 assay for assessing cell viability. (I) LDH assay for measuring cellular cytotoxicity levels. (J–L) Detection of intracellular ferrous ion, ROS, and lipid peroxidation levels using FerroOrange, DCFH‐DA, and Liperfluo probes, respectively. AOD, average optical density; ROS, reactive oxygen species; Scale bar, 200, 100 and 50 μm; **p < 0.01; ***p < 0.001; ****p < 0.0001.

As previously mentioned, the MAPK signalling pathway was significantly enriched in the hindgut region (Figure 3A). Following si‐Rack1 transfection in IEC‐6 cells, a MAPK signalling pathway PCR array was used to identify downstream molecules influenced by *Rack1* at the mRNA level. Using a threshold of |log_2_FC| ≥ 2 and *p* < 0.05 for PCR array differential gene screening, we observed a statistically significant elevation of P38δ (*Mapk13* encodin*g*) in the si‐Rack1 group (Figure 6A,B). Western blot analysis revealed an increase in phosphorylated P38 levels after Rack1 knockdown (Figure 6C,D), suggesting that interference with Rack1 enhances P38 phosphorylation, thereby activating the P38‐MAPK signalling pathway in IEC‐6 cells. Subsequently, a ferroptosis PCR array was used to screen for genes exhibiting mRNA level changes between the si‐Rack1 and doramapimod‐treated groups, revealing a total of seven genes (Figure 6E). Furthermore, the expression abundance data of these seven genes, in conjunction with existing research reports, identified *Nqo1* as a downstream molecule for further investigation. Western blotting analysis revealed that doramapimod treatment reversed the reduction in Nqo1 protein levels induced by Rack1 knockdown (Figure 6F). Additionally, the decrease in Gpx4 observed in the si‐Rack1 group was inhibited by doramapimod and restored by 2‐HBA treatment (Figure 6G).

### 3.10 Rack1 mediates ferroptosis in intestinal epithelial cells through P38 and Nqo1

Concurrent with the knockdown of Rack1 in IEC‐6 cells, rescue experiments were conducted using doramapimod and 2‐HBA. The results revealed that, compared with Rack1 knockdown alone, the addition of doramapimod and 2‐HBA led to an increase in cell viability and a decrease in relative LDH content (*p* < 0.05; Figure 6H,I). Furthermore, the intracellular ferrous ion concentration decreased, along with a reduction in ROS and lipid peroxidation levels, following the addition of doramapimod and 2‐HBA (*p* < 0.05; Figure 6J–L). These findings suggest that Rack1 modulates the intracellular iron content and lipid peroxidation through the P38 signalling pathway and Nqo1, thereby mediating ferroptosis in intestinal epithelial cells.

## 4 DISCUSSION

**FIGURE 7:**
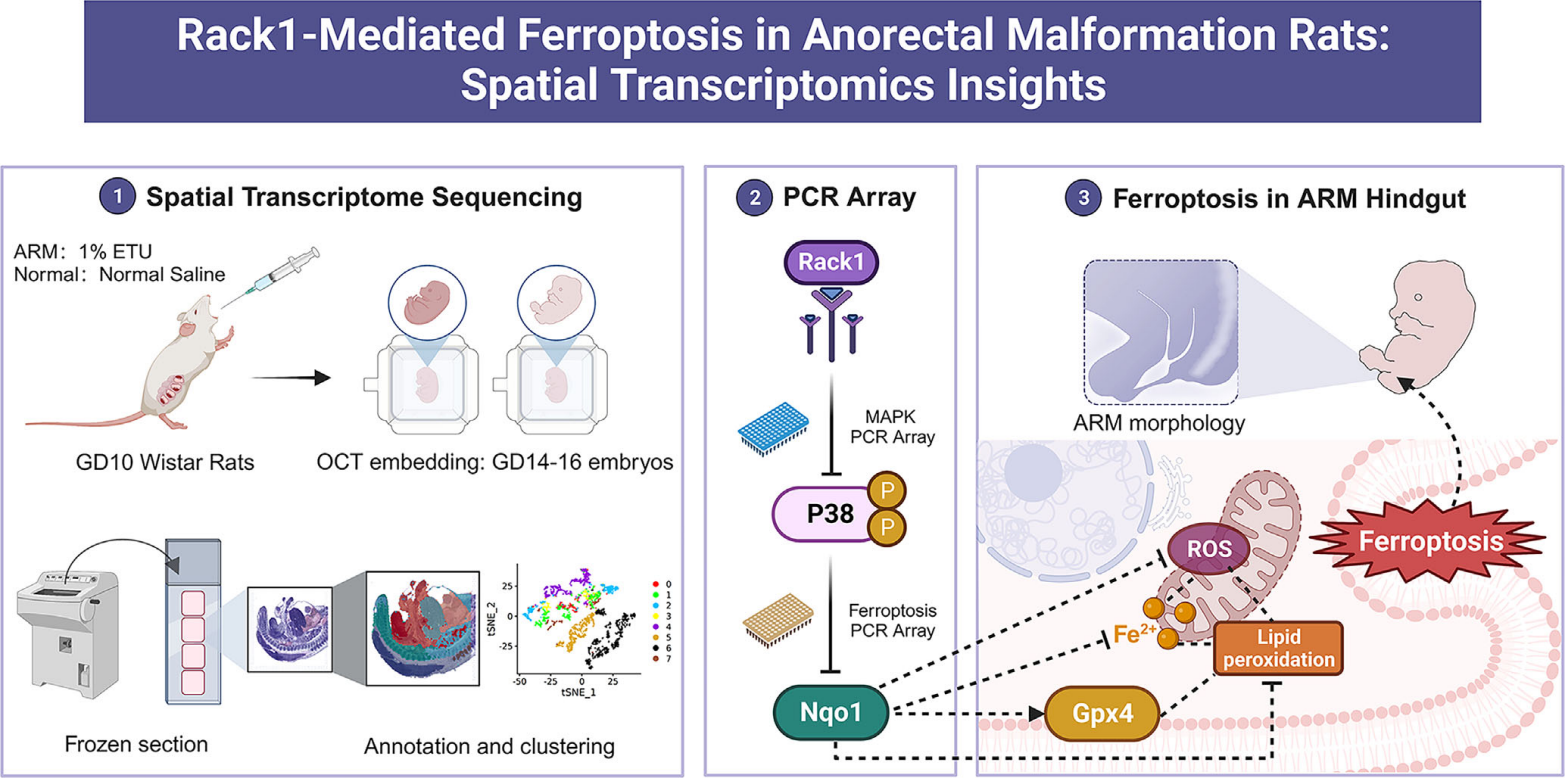
Graphic Summary. In this study, the ARM rat model was prepared by administering 1% ETU to pregnant Wistar rats via gavage on GD 10. Frozen embedding, sectioning, tissue permeation, and library construction were performed on normal and ARM rat embryos on GDs 14–16 to complete spatial transcriptomic sequencing. Anatomical annotation and dimensionality reduction clustering were conducted in the cloacal region. Through bioinformatics analysis and subsequent PCR arrays screening, it was observed that the *Rack1* gene was downregulated in the ARM hindgut on GDs 15 and 16. The downregulation of Rack1 enhanced P38 phosphorylation, which further suppressed the expression of downstream Nqo1 and Gpx4, regulating cellular iron content and lipid peroxidation levels, ultimately leading to ferroptosis in intestinal epithelial cells and contributing to the development of ARM. ARM, anorectal malformation; ETU, ethylenethiourea; GD, gestational day.

In this study, spatial transcriptomic sequencing provided site‐specific gene expression data from the cloacal region, revealing new candidates, such as *Rack1*, that might contribute to ARM pathogenesis. Subsequently, the potential function of the MAPK signalling pathway in the onset of ARM was unveiled. Lastly, we identified ferroptosis in the ARM hindgut and explored the mechanism by which *Rack1* regulates ferroptosis in the intestinal epithelium, providing novel insights into the abnormal programmed cell death mechanisms associated with ARM hindgut development. Figure 7 provides a graphical summary of the results of this study.

The expression of five hub genes in the hindgut was validated via immunofluorescence staining. Among these genes, *Uba52* deficiency results in abnormal embryonic nuclear morphology in pigs, while *Tpt1* deficiency increases apoptosis and affects central nervous system development in mice.^18^, ^19^ Their roles in ARM pathogenesis remain unknown, making them potential novel target molecules in ARM research. Rack1, the primary molecule examined in this study, is an evolutionarily conserved scaffold protein belonging to the WD40 repeat protein family. It interacts with various proteins, including kinases, ion channels, membrane receptors, G proteins, IP3 receptors, apoptosis‐related molecules and ribosomal structural proteins.^20^, ^21^ In recent years, Rack1's involvement in development‐related diseases has gained attention. A specific knockout of Rack1 in mid‐embryonic neural stem cells using hGFAP‐Cre leads to severe brain and cerebellum developmental defects in mutant mice, resulting in ataxia and balance deficits.^17^ In embryonic pigs with low body weight, reduced Rack1 levels may impair placental folding by inhibiting trophoblast cell proliferation and migration.^22^ In intestinal diseases, Rack1 regulates E‐cadherin endocytosis induced by Src and growth factors, maintaining intestinal epithelial cell connection stability.^23^ Rack1‐deficient mouse models exhibit compromised normal intestinal barrier function.^24^ Additionally, Rack1 loss in the intestine affects crypt cell proliferation and compromises differentiation into intestinal epithelial cells, goblet cells and enteroendocrine cell lineages.^25^ Finally, Rack1 also functions as a protein adaptor in the MAPK, protein kinase C and Src signalling pathways. For example, it can negatively regulate the translation of tyrosine phosphatases in the MAPK pathway, thereby modulating stress responses.^26^ The multifunctionality and crucial regulatory role demonstrated by Rack1 highlight its potential as a therapeutic target for diseases.

The MAPK signalling pathway is widely involved in cellular processes, such as proliferation, division, apoptosis, and various biochemical reactions. Its activation involves a typical three‐tiered enzyme cascade.^27^, ^28^ The PROGENy algorithm revealed significant enrichment of the MAPK signalling pathway, particularly in the hindgut region. This finding is consistent with a previous study that used whole transcriptome sequencing to identify differentially expressed circRNAs in the cloaca, which were also linked to the MAPK signalling pathway.^29^ Our investigation revealed a higher abundance of Mapk3, Mapk6 and Mapk14 in the urethra and hindgut, which are members of the Erk and P38 families.^30^ Notably, P38 is implicated in a wide range of stress‐induced cellular responses and plays a pivotal role in mouse primitive endoderm differentiation and rat synaptic development.^31^, ^32^, ^33^ Hence, this discovery provides new insights for further exploration into the role of the MAPK signal in cloacal development.

Programmed cell death is a focal point in cloacal research. The prevailing theory suggests that early anorectal development entails substantial apoptosis of URS tip epithelial cells; this process displays distinct temporal and spatial distribution patterns.^34^, ^35^ Previous studies by Qi et al.^36^, ^37^ indicated that apoptotic cells were present on the ventral side of the urogenital sinus and at the AM during GDs 14–16, suggesting the involvement of apoptotic cells in hindgut regression, urethra–hindgut separation and AM rupture. These findings highlight the potential contribution of aberrant programmed cell death to ARM pathogenesis. Ferroptosis is a form of programmed cell death, and its key molecule Gpx4 can utilise glutathione to convert harmful lipid peroxides into harmless lipid hydroxy compounds, thereby negatively regulating the onset of ferroptosis.^38^, ^39^ An appropriate iron concentration during pregnancy is essential for embryonic development, and an imbalance of iron homeostasis can affect the levels of ROS and oxidative stress, leading to diseases during pregnancy.^40^, ^41^ This study observed a reduction of Gpx4 in the ARM hindgut, leading to the investigation of the role of ferroptosis in the development of ARM.

Following *Rack1* knockdown, we observed mitochondrial alterations associated with ferroptosis in intestinal epithelial cells, accompanied by increased intracellular levels of iron, ROS, and lipid peroxidation products. Screening with a MAPK pathway PCR array and protein‐level detection revealed that Rack1 enhances P38 phosphorylation, similar to its role in multiple myeloma cells.^42^ Subsequently, specific genes identified through the ferroptosis gene PCR array (*Nox4*, *Heph*, *Nox1* and *Slc40a1*) were excluded because of their extremely low abundance in hindgut tissues or known interactions with the P38 signal. For example, P38 increases *Ncoa4* transcription, leading to intracellular iron overload and ROS elevation in periodontitis.^43^ In addition, phosphorylated P38 upregulates HUR (*Elavl1*), exacerbating chronic kidney injury induced by trimethylamine‐*N*‐oxide in rats.^44^ *Nqo1*, a downstream target gene of *Nrf2*, is a crucial antioxidant enzyme that reduces oxidised ketones to quinones, safeguarding cells from oxidative stress‐related damage.^45^ After Rack1 knockdown, we observed diminished levels of the Nqo1 and Gpx4 proteins in intestinal epithelial cells, resulting in increased intracellular oxidative stress, which was rescued by doramapimod and 2‐HBA. Cetuximab enhances Rsl3‐induced ferroptosis in colon cancer by activating the P38 pathway and inhibiting the Nrf2/HO‐1 axis.^46^ However, it remains unclear whether Nrf2 is involved in the pathogenesis of ARM and whether Nqo1 collaborates with Gpx4 to regulate lipid peroxidation levels during ARM development, necessitating further experimental research.

Despite the valuable insights provided by our study, it also has a few limitations. First, spatial transcriptomic sequencing does not yield gene expression data at the single‐cell level, which may lead to interference from multiple cells under each spot. Future studies will have to utilise higher‐resolution spatial sequencing methods or combine them with single‐cell sequencing to comprehensively and accurately reveal the interrelationships between cell clusters and gene expression during cloacal development. Second, ferroptosis in ARM embryos may be simultaneously regulated by multiple pathways, and differences may exist owing to variations in gestational timing and embryo susceptibility. However, the interplay between these molecular pathways requires further investigation. Finally, this study was based on an ARM rat model and may not precisely represent the genomic information and biological phenotypes of ARM in humans or other species. Future studies are needed to test the veracity of our results between species.

## 5 CONCLUSIONS

This study utilised spatial transcriptomic technology to sequence normal and ARM rat embryo samples during GDs 14–16. We identified new hub genes with high connectivity in the hindgut region, namely *Rack1*, *Uba52*, *Tpt1*, *Npm1* and *Eef1b2*. In contrast, we observed a significant enrichment of the MAPK signalling pathway and differential expression of Gpx4 in the hindgut region. Notably, *Rack1* exhibited decreased expression in the ARM hindgut on GDs 15 and 16, elevating the intracellular iron, ROS and lipid peroxidation levels through the P38/Nqo1/Gpx4 axis, and ultimately inducing ferroptosis in intestinal epithelial cells and potentially influencing ARM hindgut development. These findings enhance our understanding of ARM pathogenesis and have implications in advancing research on prenatal diagnosis and therapeutic strategies for ARM.

## AUTHOR CONTRIBUTIONS

All of the authors have read and approved the final manuscript. Conceptualisation, methodology and writing of the original draft were completed by C.Y.W. and M.Y.L. Data curation, supervision and formal analysis were performed by S.T.L. and Z.W.Y. Software and validation were handled by X.G.W., N.X.D. and S.Y.L. Funding acquisition, project administration, resources and editing were managed by B.L., A.P., X.B.T. and Y.Z.B.

## FUNDING INFORMATION

This work was supported by the National Natural Science Foundation of China (Grant Nos. 82070530 and 82170530) and the Outstanding Scientific Fund of Shengjing Hospital (Grant No. Me56).

## CONFLICT OF INTEREST STATEMENT

The authors declare that they have no competing interests.

## Data Availability

The data that support the findings of this study are available from the corresponding author upon reasonable request.
